# WHAT FACILITATES INFORMAL SUPPORT AMONG COMMUNITY-DWELLING OLDER PEOPLE? THE ROLE OF CARING INITIATIVES

**DOI:** 10.1093/geroni/igae098.3030

**Published:** 2024-12-31

**Authors:** Wenran Xia, Jeroen van Wijngaarden, Martina Buljac-Samardzić, Robbert Huijsman

**Affiliations:** Erasmus University Rotterdam, Rotterdam, Zuid-Holland, Netherlands; Erasmus University Rotterdam, Rotterdam, Zuid-Holland, Netherlands; Erasmus University Rotterdam, Rotterdam, Zuid-Holland, Netherlands; Erasmus University Rotterdam, Rotterdam, Zuid-Holland, Netherlands

## Abstract

**Background:**

In the context of accelerated global ageing and cutbacks in healthcare budgets, older people are encouraged to provide informal support to each other within their communities. However, the mechanism facilitating such informal support among older people is still not clear. This study aims to address this gap by investigating the perspectives and experiences of various stakeholders involved in initiatives aimed at stimulating informal support in the community setting. Method: A multiple case study was conducted in five initiatives in the Netherlands focused on facilitating informal support for older people. Semi-structured interviews were conducted with different stakeholders, including older residents who are informal support givers and receivers as well as coordinators and managers of the initiatives. A total of twenty-four participants were interviewed in twenty-one interviews. An inductive-deductive thematic analysis approach was conducted for the qualitative data.

**Results:**

Analysis revealed that older people are facilitated to participate in the community-based informal support when they have abilities, are being motivated, and have opportunities to participate in informal support. Social cohesion in the community strengthens this mechanism. Additionally, community-based formal organizations contribute to stimulate on both the individual and community levels. Conclusions: This study explores the mechanisms facilitating informal support among community-dwelling older people. It emphasizes the combination of factors at the individual, community, and organizational levels. Relevant policies and practice should be formulated with consideration of these multi-level factors.

